# Isolated Cutaneous Langerhans Cell Histiocytosis Presenting in an Adult Male

**DOI:** 10.7759/cureus.9861

**Published:** 2020-08-19

**Authors:** Lisa F Fronek, Hailey Grubbs, David W Dorton, Richard Miller

**Affiliations:** 1 Dermatology, Hospital Corporation of America / University of South Florida Morsani College of Medicine: Largo Medical Center Program, Largo, USA; 2 Dermatology, Broward Health Medical Center, Fort Lauderdale, USA

**Keywords:** langerhans cell histiocytosis, histiocytosis x, myeloid dendritic cells, dermatopathology

## Abstract

Langerhans cell histiocytosis (LCH) is an infrequent clonal proliferative disorder of myeloid dendritic cells. It has a wide variety of cutaneous manifestations and retains the possibility of systemic implications. Because LCH is predominantly a disease of childhood, there are well-established clinical definitions, as well as guidelines regarding workup and treatment, in the context of pediatric disease. Here we present a case of isolated cutaneous LCH in an adult male, followed by a discussion of our diagnostic plan and treatment course. The patient exhibited a small, excoriated, yellow papule on his inferior forehead during a skin examination. The specimen underwent tangential shave biopsy; histopathologic evaluation with appropriate immunohistochemical staining confirmed a diagnosis of cutaneous LCH. After thorough investigation via serologic and imaging diagnostics, we confirmed isolated cutaneous disease. The patient underwent wide local excision (WLE) with no evidence of recurrence. It is crucial to appropriately screen all patients diagnosed with cutaneous LCH for internal organ involvement. The authors aim to highlight the need for further investigations to ultimately dictate standardized management and treatment for isolated cutaneous LCH in the adult population.

## Introduction

Langerhans cell histiocytosis (LCH), previously termed histiocytosis X, is a rare inflammatory, neoplastic condition of myeloid dendritic cells that express an immunophenotype positive for CD1a, langerin (CD207), S100, and cytoplasmic Birbeck granules [1, 2]. Based on similarities in immunophenotypic markers, LCH was thought to arise from skin Langerhans cells, the primary antigen-presenting cells in the epidermis. Newer gene expression analyses demonstrated that the neoplastic cells in LCH derive from immature myeloid precursor cells from the bone marrow [1-3]. The pathogenesis of LCH was presumed to be viral or immunologic; however, the finding that 55-60% of LCH specimens contain the BRAF V600E mutation determined its neoplastic status. The BRAF protein is a serine/threonine kinase that participates in the mitogen activation protein kinase pathway (RAS-RAF-MEK-ERK), thus elucidating the derivation of this disease and suggesting a possible therapeutic role for BRAF inhibitors [2-4].

Historically, LCH is divided into four distinct syndromes: Letterer-Siwe disease, Hand-Schüller-Christian disease, eosinophilic granuloma, and congenital self-healing reticulohistiocytosis (Hashimoto-Pritzker disease). In clinical practice, many patients do not fit precisely into a particular entity, frequently exhibiting overlapping manifestations [1]. Clinically, LCH is further categorized based on the extent of disease (single or multisystem) and involvement of at-risk organs.

LCH is primarily a disease of childhood, with an incidence of approximately five per every million children and cutaneous involvement in as many as 40% of cases [1,5]. In comparison, the incidence of LCH in adults is one to two per million [6]. Clinical manifestations of LCH vary; it may present in single or multiple organ systems, single or multiple sites within one specific organ system, or in risk organs, such as the skeletal system, liver, spleen, and skin [1]. Cutaneous presentations range from light brown, yellow-orange, or violaceous macules, papules, or plaques. LCH is known to mimic common cutaneous disorders such as seborrheic dermatitis or atopic dermatitis. It may also have a varicelliform appearance or present as recalcitrant diaper dermatitis.

As mentioned previously, LCH in adults is a rare clinical entity, especially when confined to the skin. Here we present a case of isolated cutaneous LCH in an adult male. The purpose of this report is to discuss a rare clinical entity and the screening, treatment and surveillance guidelines for LCH in adults.

## Case presentation

**Figure 1:**
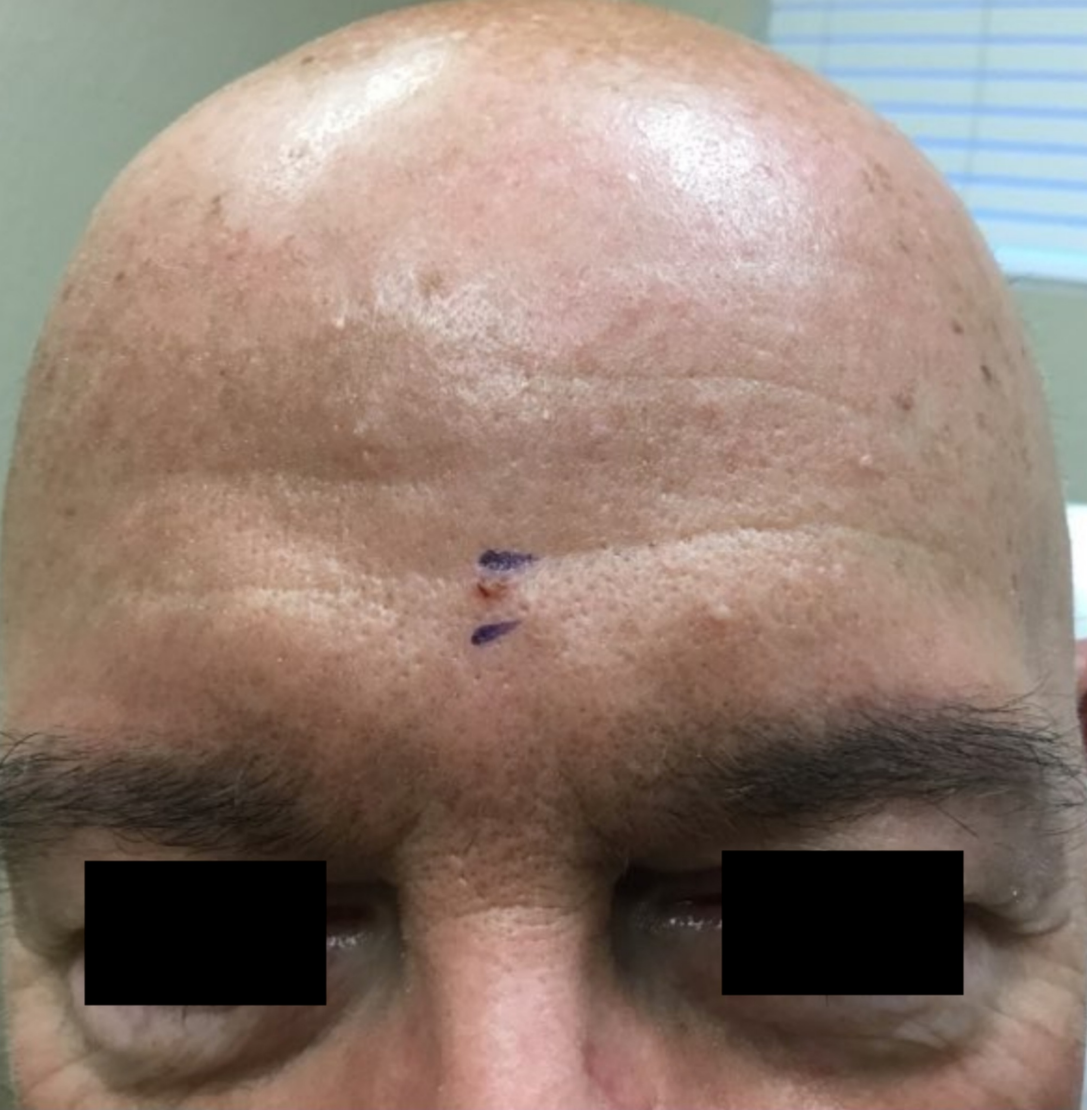
A small, yellow to light brown, keratotic papule in the center inferior portion of the forehead of a Caucasian male.

A 65-year-old Caucasian male with a history of hypertension and no prior cutaneous malignancy presented to our dermatology clinic with a chief complaint of an asymptomatic, keratotic, yellow papule on the inferior mid-forehead, first noted approximately six weeks prior to examination (Figure 1). There were no surrounding telangiectasias or erythema. The papule was slightly excoriated yet remained intact; the patient denied significant pruritus. Tangential shave biopsy of the lesion was performed.

**Figure 2:**
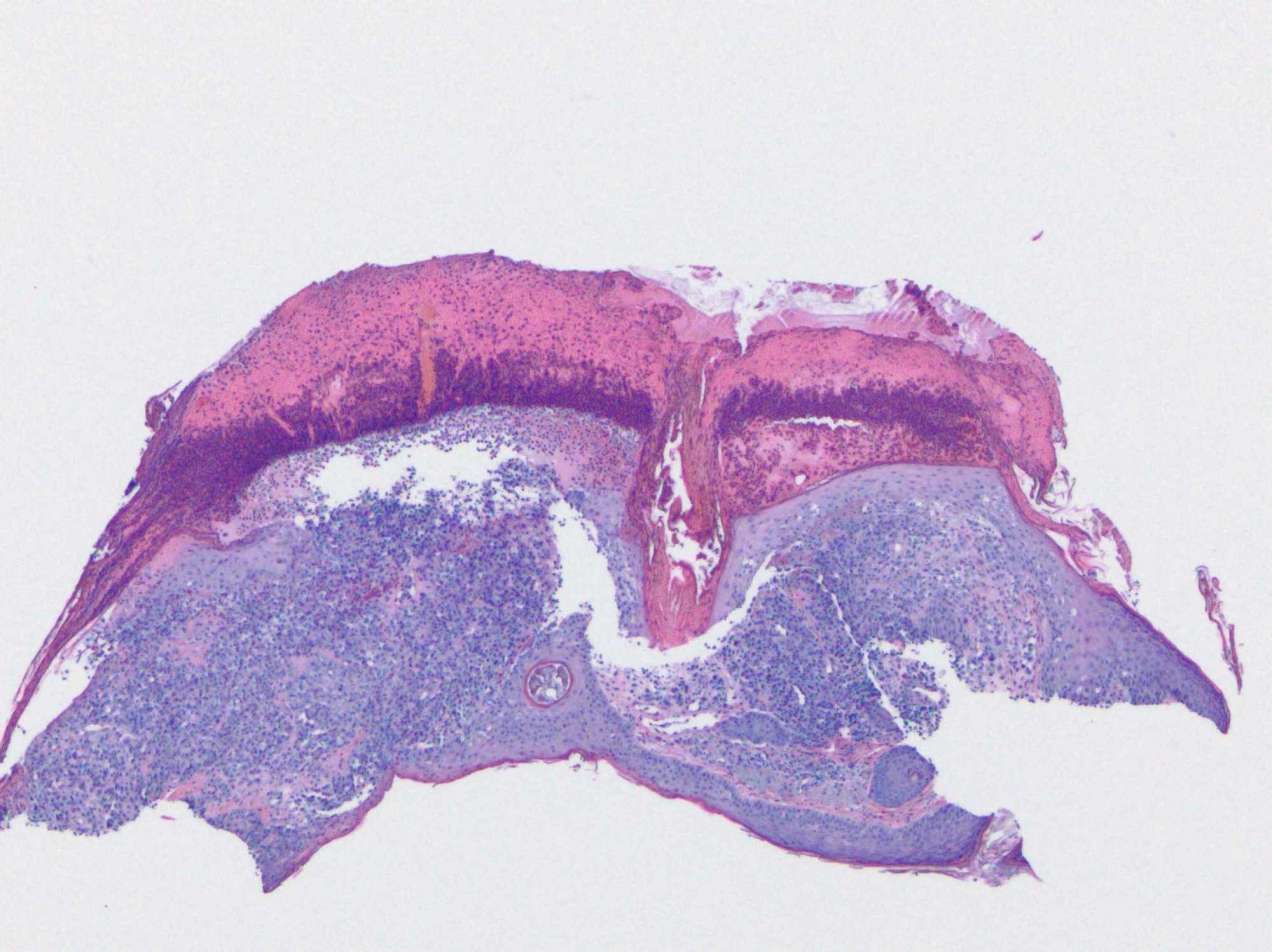
A tangential shave biopsy of the area of concern was submitted for hematoxylin and eosin (H&E) stain. This revealed a predominant dermal infiltrate with significant amount of neutrophilic crust overlying the epidermis (4X).

**Figure 3:**
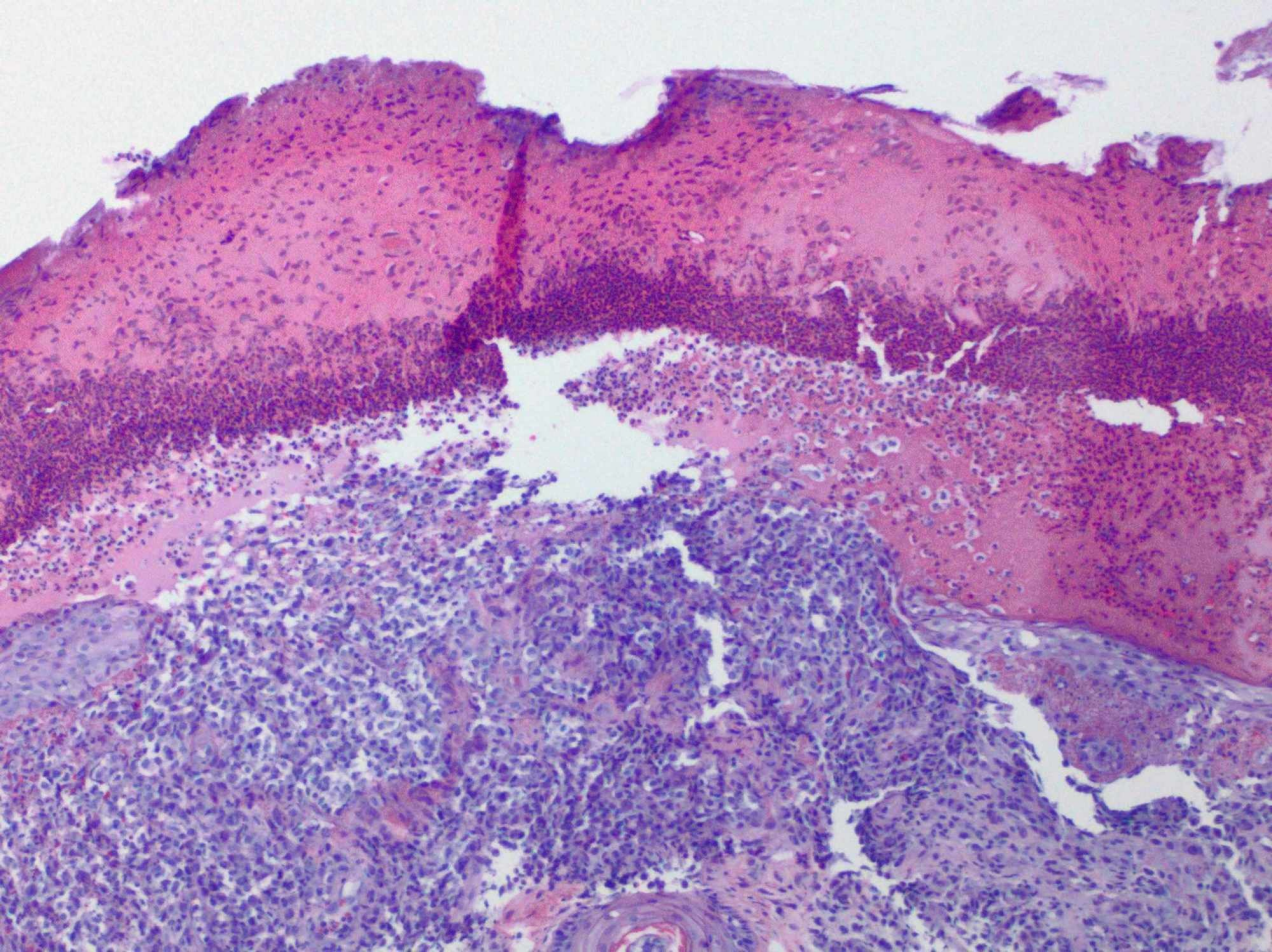
The polymorphous infiltrate contained atypical appearing monocytes, neutrophils, and eosinophils (10X).

**Figure 4:**
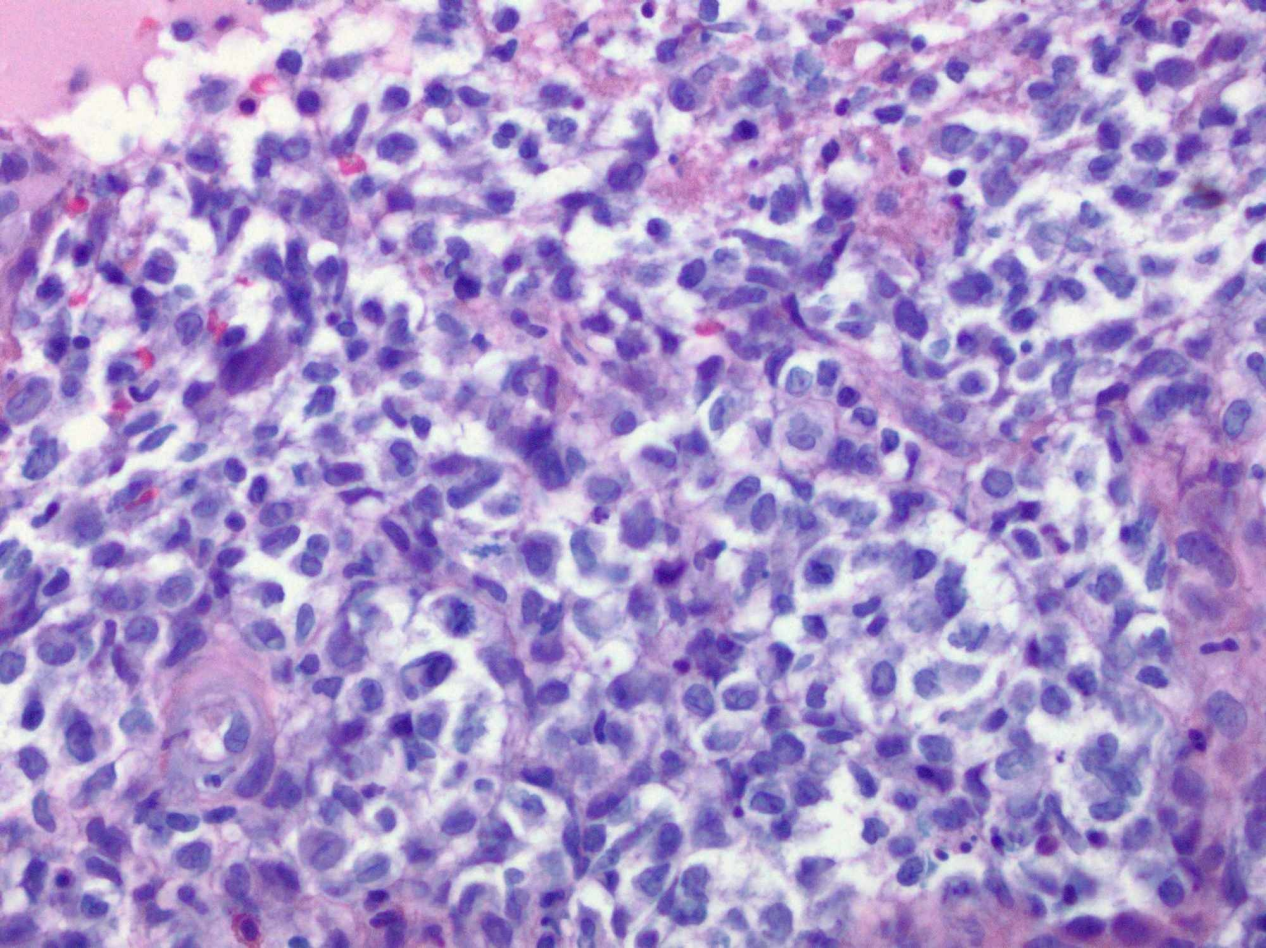
Focus of heavily cellular atypical monocytic population throughout the papillary dermis (40X).

**Figure 5:**
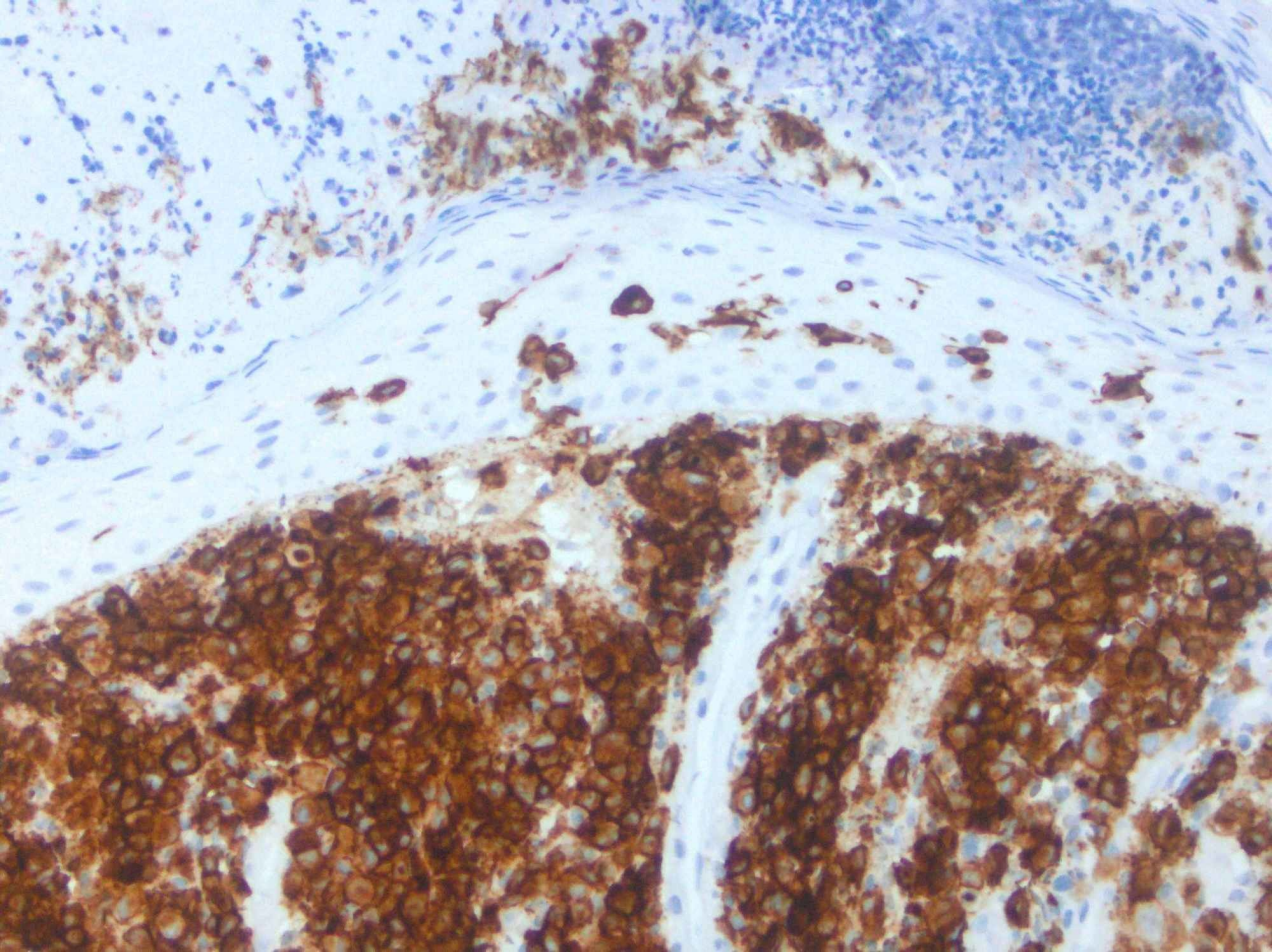
Appropriate immunohistochemical (IHC) stains performed of lesional cells shows positivity for CD1a.

**Figure 6:**
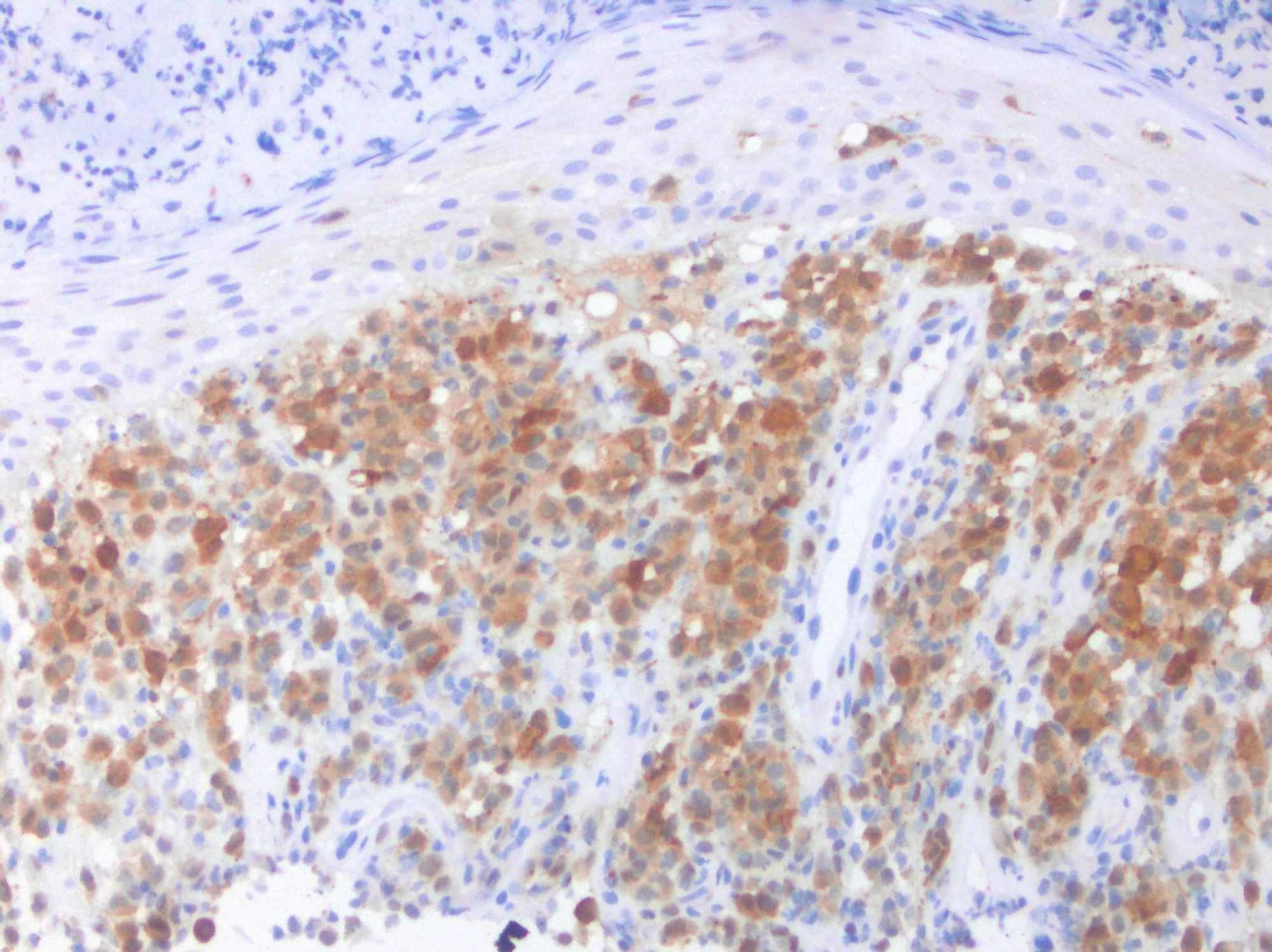
IHC performed of lesional cells shows positivity for S100.

**Figure 7:**
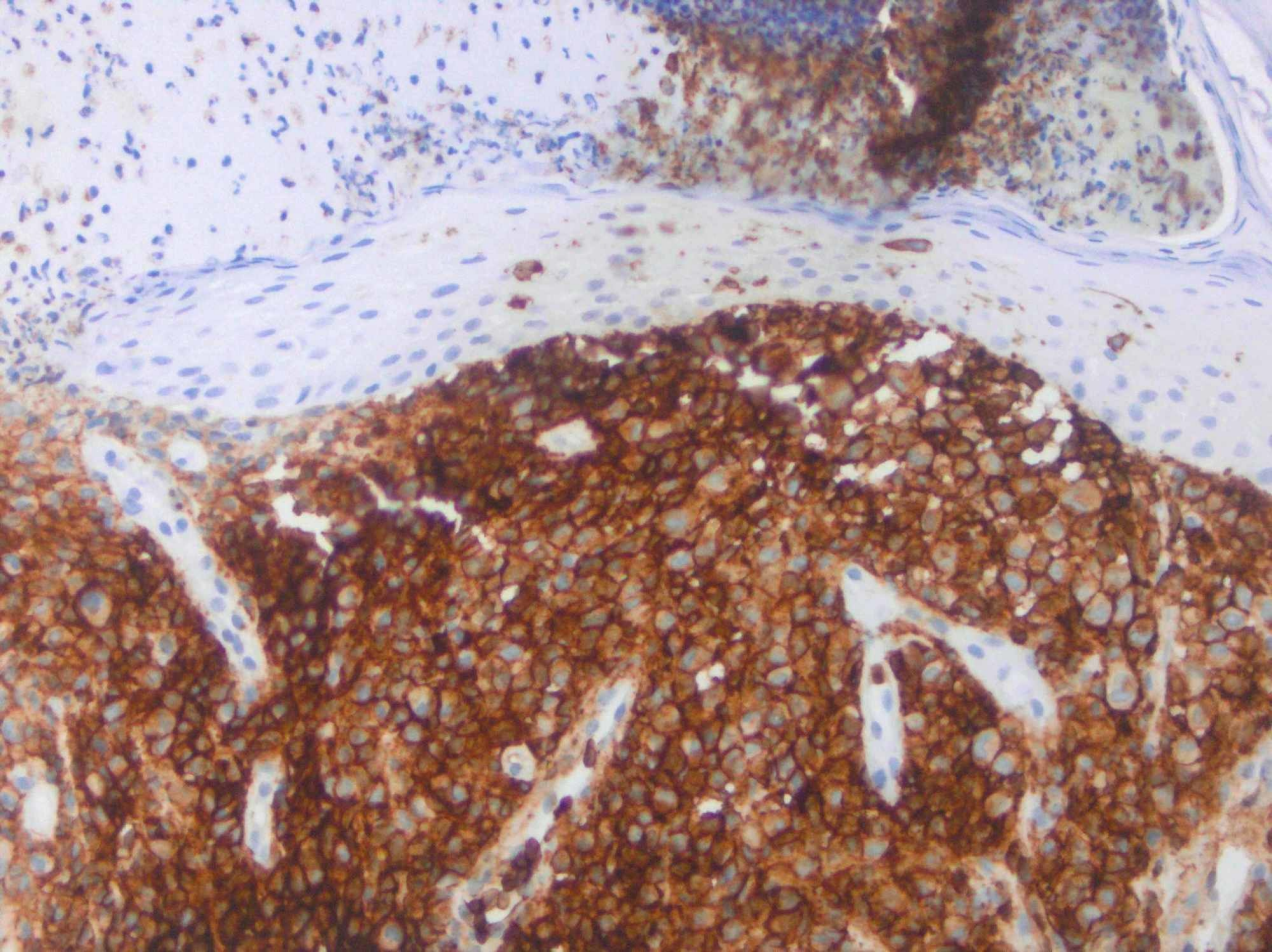
IHC performed of lesional cells shows positivity for CD45.

**Figure 8:**
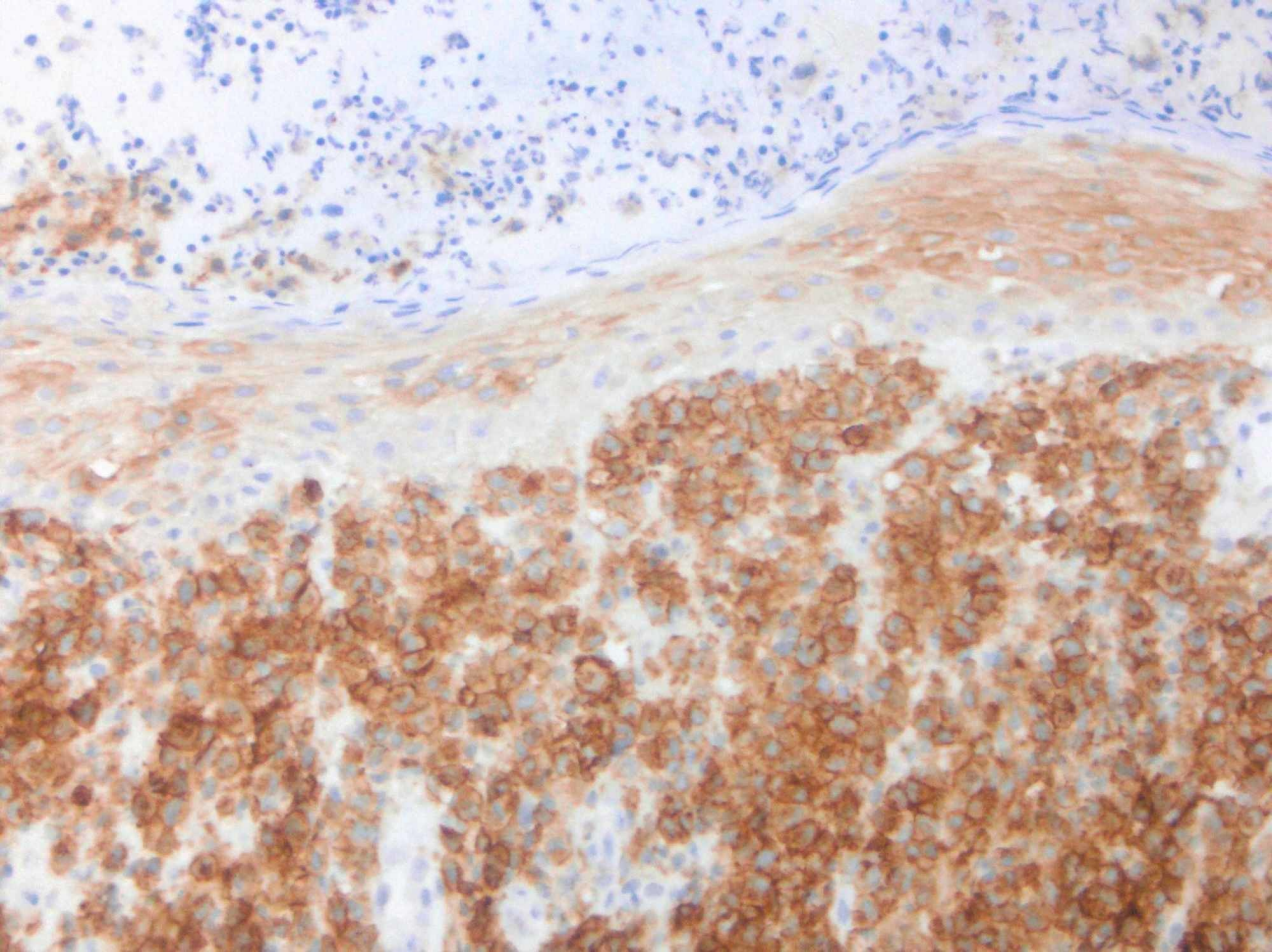
IHC performed of lesional cells shows positivity for CD4.

**Figure 9:**
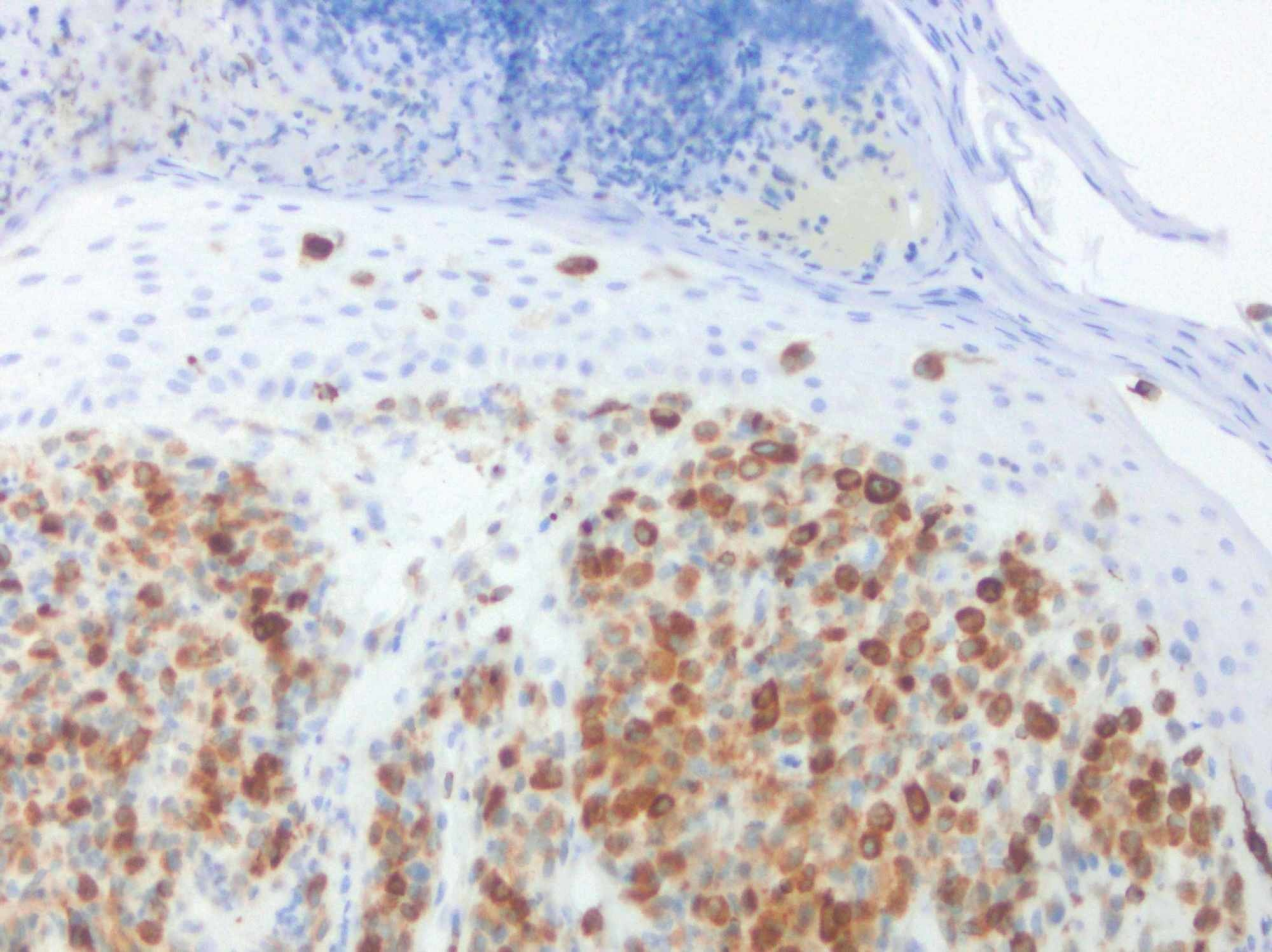
IHC performed of lesional cells shows positivity for BCL-2.

Histopathologic report revealed a papule with predominantly dermal infiltrate, and an overlying neutrophilic crust (Figure 2). The dermal polymorphous infiltrate contained atypical mononuclear cells, neutrophils, and eosinophils with the additional heavily neutrophilic crust above the epidermis (Figure 3). Ultimately, this atypical mononuclear infiltrate (Figure 4) demonstrated lesional cells positive for CD1a (Figure 5), S100 protein (Figure 6), CD45 (Figure 7), CD4 (Figure 8) and BCL-2 (Figure 9), consistent with LCH. Additional stains for SOX-10, Melan-A and myeloperoxidase were negative.

On further discussion, the patient denied fevers, chills, fatigue, weight changes, arthralgias, myalgias, headaches, polyuria and polydipsia. After referral to hematology/oncology, he underwent brain magnetic resonance imaging (MRI) with and without contrast and skull base-to-thigh positron emission tomography (PET)/computed tomography (CT). Brain MRI depicted mild chronic microvascular changes in the white matter, unchanged from a prior study. PET/CT demonstrated no evidence of fluorodeoxyglucose (FDG) avid malignancy with no hypermetabolic osseous lesions nor FDG avid lymphadenopathy. After approval by hematology/oncology, the patient underwent wide local excision (WLE) with 0.5 cm surgical margins. Histopathological analysis of the surgical specimen revealed reparative changes without evidence of residual LCH.

## Discussion

LCH is a rare clonal proliferative disorder derived from myeloid dendritic cells. This entity is defined by the presence of CD1a, S100, langerin (CD207) staining Langerhans-like cells and Birbeck granules [3]. While LCH can occur at any age, it most commonly presents between the ages of one to three years. In childhood, there is a slight male predominance; in adulthood there is a minimal female predominance [2]. The incidence of LCH in children is about five per million cases; in contrast, adult LCH has an estimated incidence one to two cases per million per year [7]. LCH can be categorized into four syndromes: Letterer-Siwe disease, Hand-Schüller-Christian disease, eosinophilic granuloma, and congenital self-healing reticulohistiocytosis (Hashimoto-Pritzker disease). Considering their origin from dendritic cells and key molecular findings, Emile et al. proposed a new classification for histiocytic disorders, defining five types: Langerhans, cutaneous and mucocutaneous, malignant, Rosai-Dorfman, hemophagocytic lymphohistiocytosis, and macrophage activation syndrome [8]. Within this schema, LCH belongs in the Langerhans group, along with Erdheim-Chester disease and indeterminate cell histiocytosis, based on its activating mutation in the MAPK pathway [1].

Clinical classification of LCH begins with division into single- or multi-system disease. Bone is the most common site of involvement, followed by the integumentary system [9]. LCH involves the skin in 25-40% of cases. In patients under two years of age, cutaneous disease is both the most common presenting finding and an ominous sign, as 87-93% of patients with skin disease have systemic involvement [1]. Among involved organ systems, at-risk locations include liver, spleen and bone marrow. Cutaneous LCH is typically associated with other organ involvement; isolated cutaneous disease accounts for a mere 2% of total cases [1].

Cutaneous manifestations are variable. LCH can mimic seborrheic dermatitis, eczematous dermatitis or manifest as solitary or grouped papules, nodules, or ulcerations [1]. Pruritus is common feature described by patients. Regardless of the primary lesion, LCH presents most often on the trunk, head and neck, followed by extremities, intertriginous sites, buttocks, and occasionally the oral mucosa. When presenting in the skin, diagnosis is made when biopsy shows mononuclear cells with characteristics of Langerhans cells (Figures 2-4): CD1a, langerin, S100 positive staining, and Birbeck granules on ultrastructural examination. Our patient had a solitary, small, asymptomatic papule on the inferior portion of his forehead. Histologically, this lesion was suspicious for an atypical monocytic infiltrate. IHC stains were positive for CD1a and S100 (Figures 5, 6). While the most specific IHC stain, langerin (CD207) was not performed, there was enough evidence to support the diagnosis of LCH. Appropriate negative stains for myeloperoxidase ruled out a leukemic process, as well as negative Melan-A and SOX-10, which ruled out an atypical melanocytic process.

All patients should undergo a thorough history and physical examination to assess the extent of disease. Attention is directed to skin, lymph nodes, ears, oral cavity and mucosa, skeletal system, lungs, thyroid, liver, spleen and central nervous system. Constitutional symptoms (fevers, chills, fatigue, weight loss, and lymphadenopathy) are sought and may indicate bone marrow or lymph node involvement. Polyuria or polydipsia could suggest pituitary involvement, while headaches, nystagmus, tinnitus might indicate cranial infiltration affecting ocular or auricular structures. A complete blood count (CBC), comprehensive metabolic panel (CMP), skeletal survey, chest radiography (CXR) and sonography of the liver and spleen are recommended [10]. Edelbroek et al. reported 18 patients with LCH initially presenting with skin lesions and an additional 74 cases from a literature search spanning 1976-2011. They argued for a minimum workup of CBC, CMP, CXR, bone scintigraphy, and bone marrow biopsy to screen for systemic LCH [6]. Additional testing and imaging are contingent on organ-specific symptoms. Our patient denied any constitutional symptoms such as fevers, chills, weight loss. He also denied abdominal pain, specific bone pain or arthralgias, polydipsia or polyuria. Additionally, the patient's most recent CBC and CMP showed no abnormalities. He then underwent brain MRI and PET/CT, based on recommendations made by the patient’s hematologist, with no significant metabolic uptake. This further confirmed his isolated cutaneous LCH.

Prognosis and treatment are contingent on the degree of systemic involvement and the presence of a BRAF mutation [4]. Due to the patient's isolated cutaneous disease and no systemic involvement, it was decided to not pursue further BRAF testing. Local therapy is the treatment of choice for single-system LCH specific to the skin or bone [3]. Local therapy may consist of either surgical excision, topical nitrogen mustard, topical tacrolimus, or phototherapy therapy [11, 12]. Furthermore, it is recommended that surgical excision be reserved to those cases of isolated cutaneous LCH with a solitary skin lesion [3]. Other groups have found success in case series utilizing systemic methotrexate or thalidomide for skin limited disease [13, 14].

After conferring with hematology/oncology and performing all appropriate laboratory and imaging diagnostic tests, it was decided that the patient undergo WLE utilizing 0.5 cm margins. Upon histological analysis of the excision specimen, there were reparative changes and an incidental dermal nevus; however, no evidence of residual LCH. The patient continues to follow with the dermatology department for routine skin examinations every six months, according to pediatric guidelines following isolated cutaneous LCH [10]. The reason for these frequent visits is to primarily assess for recurrence or systemic dissemination [15]. Another goal of periodic examinations is to screen for secondary malignancies; Edelbroek et al. highlighted that while isolated, cutaneous LCH in an adult is extremely rare, these patients have a greater than 25% risk for developing a secondary hematologic malignancy at median 41-month follow-up. This finding further argues the need for adequate surveillance guidance for patients with isolated cutaneous disease. Specific timeframes for follow-up in adult LCH patients are not established and is instead left up to the comfortability of patient and physician.

## Conclusions

LCH is an uncommon inflammatory and neoplastic condition of myeloid dendritic cells. Traditionally, it has been classified into one of four syndromes: Letterer-Siwe disease, Hand-Schüller-Christian disease, eosinophilic granuloma, and congenital self-healing reticulohistiocytosis. While LCH is predominantly seen in children, the disease is not exempt from affecting adults, albeit less frequently. It is important to adequately screen all patients diagnosed with cutaneous LCH for systemic manifestations of this disease, as this can affect management and overall prognosis.
